# Inclusive housing for people with disability: process evaluation of the ‘Down to 10 Days’ campaign

**DOI:** 10.1093/heapro/daaf082

**Published:** 2025-06-18

**Authors:** Ali Lakhani, Di Winkler, Ruth Mackenzie-Stewart, Jessica Walker, Deniz Senyel, Rwth Stuckey

**Affiliations:** School of Psychology and Public Health, La Trobe University, 360 Collins St, Melbourne, VIC 3000, Australia; The Hopkins Centre, Menzies Health Institute Queensland, Griffith University, Logan Campus, University Drive, Meadowbrook, QLD 4131, Australia; Research and Innovation, Summer Foundation, Box Hill, VIC 3128, Australia; Living with Disability Research Centre, La Trobe University, Kingsbury Drive, Bundoora, VIC 3086, Australia; School of Psychology and Public Health, La Trobe University, 360 Collins St, Melbourne, VIC 3000, Australia; Research and Innovation, Summer Foundation, Box Hill, VIC 3128, Australia; School of Psychology and Public Health, La Trobe University, 360 Collins St, Melbourne, VIC 3000, Australia; School of Psychology and Public Health, La Trobe University, 360 Collins St, Melbourne, VIC 3000, Australia

**Keywords:** process evaluation, disability, housing, self-directed funding, self-directed funding

## Abstract

The *Down to 10 Days* campaign was an advocacy initiative aimed at reducing long delays in housing approvals under Australia’s National Disability Insurance Scheme (NDIS), a federal self-directed funding program for people with disability. Delays in securing appropriate housing often resulted in prolonged hospital stays or inappropriate placements, significantly impacting individuals’ well-being and independence. The campaign, led by a coalition of disability and advocacy organizations, sought to streamline NDIS approval processes, targeting a reduction in decision times to 10 days. A process evaluation systematically examines a program or campaign's implementation, assessing whether activities were delivered as intended and identifying factors influencing success. It is particularly valuable in advocacy, offering insights into campaign effectiveness, stakeholder engagement, and short- and long-term outcomes. A process evaluation utilizing Kotter’s eight-Step Change Model, incorporating document analysis (*n* = 42) and semi-structured interviews with key stakeholders (*n* = 6), was undertaken to assess the campaign’s implementation and effectiveness. The use of clear messaging, targeted political advocacy, and public engagement strategies contributed to the campaign’s reach and influence. Key short-term successes included heightened awareness, improved government transparency regarding NDIS decision timelines, and commitments to process improvements. This evaluation underscores the importance of strategic advocacy, coalition-building, and evidence-based messaging in driving policy change. The findings provide valuable insights for future campaigns seeking to enhance disability services and social policy reforms.

Contribution to Health Promotion

- This process evaluation contributes to health promotion by demonstrating the value of systematically assessing advocacy initiatives to inform future public health campaigns.
- By identifying key factors influencing campaign effectiveness—such as stakeholder engagement, coalition-building, and evidence-based messaging—this evaluation highlights how advocacy can drive policy change and service improvements.
- The findings emphasize the importance of real-time feedback and adaptive strategies in advocacy efforts, ensuring resources are directed towards impactful actions.
- These insights provide a framework for future health and social policy advocacy, supporting organizations in developing more effective, evidence-informed campaigns to promote equity and systemic change.

## BACKGROUND

Access to suitable housing ensuring health, wellbeing and safety is a determinant of health and a human right (United Nations General Assembly 1949; Office of the High Commissioner for Human Rights 1976). The United Nations Convention on the Rights of Persons with Disabilities (United Nations General Assembly 2006) highlights the right of people with disability to live independently and be included in the community. The National Disability Insurance Scheme (NDIS) is Australia’s first national government self-directed funded initiative designed to provide support to individuals with permanent and significant disabilities, their families and carers (National Disability Insurance Agency 2025). Self-directed funding for people with disability is increasingly prevalent across the western world; recipients emphasize the value of being able to access supports to best support their needs (Lakhani *et al*. 2018a, 2018b).

NDIS Specialist Disability Accommodation (SDA) policy and payments are designed to support individuals with disability by providing housing and supporting independence. A recent report investigated wait times in hospital as a result of unsuitable accommodation for ageing adults and people with disability. It confirmed that prior to June 2022, NDIS eligible people within hospital waited ∼160 days for suitable NDIS support, including accommodation, prior to discharge (Australian Medical Association 2023).

### Advocacy and process evaluations

Advocacy is central to health promotion, addressing the determinants of health and ensuring rights through systemic change (World Health Organization 1986; Bhattarai *et al*. 2020; Flavel *et al*. 2024). Process evaluation is essential for determining whether the implementation of advocacy campaigns is undertaken as initially intended and which advocacy activities have contributed to or hindered achievement of campaign outcomes (Mackenzie-Stewart and Khalil 2023). Process evaluation of advocacy campaigns provides a systematic approach to documenting how campaigns operate and progress including whether intended strategies are implemented, while assessing quality, accessibility, and acceptability of advocacy messages, utilization of resources, and any adjustments made throughout the campaign (Zhao 2020). Evidence shows advocacy campaigns can drive change in public and commercial health determinants (Freudenberg *et al*. 2009; Samuels-Dennis *et al*. 2016; Capitão *et al*. 2022). While advocacy activities frequently take place across the disability sector (Brolan *et al*. 2012; Jaiswal and Gupta 2017), often limited or no evaluation is undertaken resulting in scant evidence to inform future advocacy campaigns. This is problematic in a climate of limited resources, hindering the evidence based targeting of resources into advocacy with the potential to reach the right audience at the right time to drive change.

### The Down to 10 Days campaign

*Down to 10 Days* was an advocacy campaign backed by an alliance comprising individuals with disabilities, advocacy groups, and stakeholders in housing, health, and disability sectors. The coordination of this alliance was overseen by an Australian disability research and advocacy organization. The purpose of the *Down to 10 Days* campaign was to ensure that Australians with disability obtain timely approval for essential housing and support services funding, and in particular, streamlined and efficient NDIS processes. The campaign aimed to drastically reduce this timeframe, with the goal of achieving approval for housing and support within 10 days. Campaign participants were encouraged to sign a petition, join the alliance, and support people with lived-experience to share their stories publicly.

It is expected conducting a process evaluation of an advocacy campaign which aims to reduce wait-times for housing and support funding for people with disability can inform the strategies for use by other organizations collectively advocating for social policy change. Consequently, the aims of a process evaluation were to (i) clarify the process of the *Down to 10 Days* campaign (hereafter the Campaign) and (ii) identify effective elements of the Campaign and factors contributing to effectiveness.

## METHODS

### Approach

This study was approved by the La Trobe University Human Research Ethics Committee (HEC23229). Informed by Rosewarne *et al* (2021), Kotter’s 8-step framework (Kotter 2007), was adapted. This involved a document review (US Department of Health and Human Services 2018) and semi-structured interviews with key stakeholders. Kotter’s eight-step model, originally developed for organizational change (Kotter 2007), has since been applied in public health and advocacy (Moore *et al*. 2013; Rosewarne *et al*. 2021). The steps, under the context of creating change through public health advocacy (Kotter 2007) has been detailed in Table 1.

**Table 1.** Description of Kotter’s eight-step framework applied to public health advocacy.

Step	Description
Step 1: Establishing a sense of urgency	Public health advocacy begins by highlighting pressing health issues. Communicating the urgency of these issues broadly and dramatically to motivate communities, policymakers, and stakeholders to support change.
Step 2: Creating the guiding coalition	A powerful coalition of public health leaders, community advocates, healthcare providers, and policymakers must come together to guide advocacy efforts. This group should develop a shared commitment and work collaboratively to achieve their goals.
Step 3: Developing a change vision	A clear and compelling vision for public health improvement is crucial and should articulate the desired outcomes, such as reduced disease prevalence or improved health equity, and should be easily communicated to and understood by all stakeholders.
Step 4: Enlist a volunteer group	The vision for public health change must be communicated frequently through various channels, such as community meetings, media campaigns, and policy briefs. Leaders should embody the vision and align with stakeholders’ efforts. Building a volunteer army involves rallying individuals and organizations to actively support and participate in the advocacy efforts, ensuring sustained momentum.
Step 5: Enable action by removing barriers	Removing barriers hindering public health advocacy is essential. This involves addressing policy restrictions, funding limitations, or systemic inequities. Empowering community members, organizations, and advocates to take action towards the vision is crucial for progress.
Step 6: Generating short-term wins	Achieving short-term wins, such as passing a health policy, launching a successful public health campaign, or improving health outcomes in a specific community, is necessary to maintain momentum, motivation and commitment. These wins should be visible and directly linked to the advocacy vision.
Step 7: Sustained acceleration	Avoid declaring victory too soon after initial successes, rather use the credibility gained from short-term wins to tackle larger public health issues and embed change. Continuous advocacy efforts and attention are required to prevent regression and ensure sustained improvements in public health.
Step 8: Institute change	For public health changes to stick, they must become a part of community and organizational culture. This involves showing how new public health policies and practices have improved health outcomes and ensuring that future leaders and advocates embody the new approaches. Institutionalizing these changes ensures sustainability and continued progress.

### Recruitment

The campaign was coordinated by a national disability research and advocacy organization with extensive experience in housing, health, and policy reform for people with disability. This organization led the design and implementation of campaign activities in collaboration with a wider alliance of partners and is hereafter referred to as the ‘leading organization’.

Internal documents and stakeholder interviews were collected for the evaluation. To identify and collate internal documents, the first three steps of the document review processes outlined by the US Centers for Disease Control and Prevention (US Department of Health and Human Services 2018) and Morgan (2022) were adopted. De-identified documents and materials considered included: internal meeting minutes and presentations, board member reports and meeting minutes, project budgets, external facing advertisements, public media write-ups, and campaign project planning materials.

A purposeful sampling approach was adopted to recruit staff, and/or industry stakeholders for participation in semi-structured interviews. The social service and advocacy provider emailed relevant stakeholders (*n* = 18) with a strong understanding of the campaign and/or the issue. The email included an information sheet and consent to be contacted form. Stakeholders who completed the consent to be contacted form advised the researcher who contacted them to discuss the interview process and share the study information sheet. Participants provided written consent to the researcher.

### Data collection

The semi-structured interviews included open ended-questions informed by the most-significant change approach (Davies and Dart 2005), and Kotter’s framework (Kotter 2007). Specifically, questions aligning with Kotter's eight-step change management process were utilized. To finalize the interview protocol a question assessing perceived change was developed, aligning with the most-significant change approach. All interviews were undertaken via Zoom (Zoom Video Communications 2025), and audio recorded. All data were securely stored within the University’s cloud system.

### Data analysis

A hybrid deductive and inductive analysis approach was employed (Fereday and Muir-Cochrane 2006). Interview and documents data were deductively coded against Kotter’s eight-steps. Information included under each of these eight-steps were then inductively analysed to establish emergent themes. Using NVivo (Q. S. R. International Pty Ltd 2020), two researchers independently coded data and worked with the larger research team to reach a consensus on the main findings underpinning each of Kotter’s eight-steps.

## RESULTS

### Participants and document overview

Seven semi-structured interviews were conducted, five of which were with leaders from the leading organization, with distinct project roles, and two external senior managers working in the field of health and social care for people with disability. The mean interview length was 46 minutes. Forty-two documents were reviewed within the domains described in Table 2. Supplementary File S1 provides a Campaign Timeline and contextualizes findings.

**Table 2.** Type of documents reviewed.

Type of documents	Number of documents
Internal documents e.g. discussion papers, meeting presentations, workshop ideas and creative conversations.	15
Publications including newspaper articles about the campaign, public statements made by elected officials, and campaign reports and statements produced by health and social service organizations.	12
Public campaign documents including internal documents surrounding campaign budgeting and timelines, and external documents including campaign advertisements	8
Board related documents including presentations, documents and meeting minutes from board meetings.	7

### Presentation of findings

The combined results from the interviews and document review provide a significant understanding of the Campaign's implementation and outcomes. These results are presented under each step of the process evaluation.

### Creating a sense of urgency

This first theme addresses the work within the leading organization to develop internal momentum to pursue the identified opportunity with a common vision. The campaign was not typical of the work of the leading organization, which was described as: ‘we mostly are the quiet achiever ………we’re focused on impact, but we will do whatever it takes to have that impact and use the right tool for the right time.’ The campaign was considered the tool for the time, driven by both an underpinning understanding of the problem, and an opportunity created by the impending election. At the time of the campaign, Australia was approaching a federal election in May 2022, with disability funding and NDIS performance emerging as key issues in public and political discourse. Pre-election periods are typically a strategic time to influence policy commitments, as political parties are more responsive to public pressure and sector alignment. As one participant explained, ‘an election is a unique opportunity to set a policy agenda and make some significant asks from both sides.’

Responding to opportunity is not new to the leading organization: ‘if the opportunity comes up, we will do it. even if it’s short notice, we’ll do it’. A member of the board highlighted, previous meetings with stakeholders, including government, did not lead to results. Specifically, 1:1 meetings and advisory group discussions with the Minister and National Disability Insurance Agency (NDIA) staff had not resulted in concrete policy commitments, measurable reductions in decision timeframes, or changes in NDIA processes. Therefore, it was decided to exploit the timing and change tact: ‘we wanted to come up with a pre-election campaign that would be very specific and get traction, and an idea and a concept that we thought was going to make the biggest difference at that point in time, to the [cohort] of people with disabilities who need access to 24/7 support.’ At the February 2022 Board meeting, a paper was presented which ‘noted discussions at previous Board meetings regarding the frustration at the lack of real change on key issues affecting young people in RAC, despite our strong advocacy work over many years.’ This led to the decision to take a more public approach to the leading organization’s advocacy.

The first step was to inspire passion and purpose in others with an interest in ‘get(ting) the government on side and commit(ed) to bringing down the number of days that it takes for home and living decisions to be made for people putting in an application and it being reviewed’. This required a clear and compelling evidence-based vision. Finding data to support concerns, particularly around NDIS time taken to make decisions was quite difficult; the organizational lead staff pulled data together from a variety of sources including fast tracking analysis data from an advocacy group (see Skipsey *et al*. 2022, 2023) who: ‘had the data on how long it was taking for their clients to seek funding to be approved and then to finally move into their housing.’ This Housing Hub report (Skipsey *et al*. 2022) provided critical analysis of the NDIA decision-making timeframes, and confirmed that median internal funding decision times prior to 2022 were 97 days. Furthermore, Cubis *et al*. (2022) found that delays in the completion of Access Request Forms by hospital personnel and approvals for support and housing funds by the NDIS, in part due to a thin housing market, a lack of specialized care, and suitable accommodation for people with high care needs.

The time was right and urgency clear and evidence based, as one participant described: ‘the combination of the cost of hospital beds and the fact that our hospital beds were already under pressure because of COVID, it was quite a compelling argument for the NDIA to step up and provide more timely decisions on funding for housing and support, just because of the impact on the health system……but then we also had really strong evidence around the specific time frames and potentially a lot of evidence about what was happening in the hospitals.’ Solutions required more consistent and timely NDIS funding decisions to enable access to appropriate accommodation, the aim of the campaign being to raise awareness of this pressing issue. The opportunity presented itself—‘an opportunity to re-frame our challenges as an election promise and a win for the Coalition and Labor governments. Politicians are reluctant to spend any money—apart from during an election campaign.’ The campaign was launched with momentum to engage others and pursue this vision for constructive change.

Possibly because of the speed required to act in a limited timeframe, Steps 2, 3, and 4 of Kotter's model were integrated and somewhat overlapping, and while these are addressed separately, they occurred rapidly and at times concurrently.

### Build a guiding coalition

The second theme addresses the development of a coalition, led by the leading organization, to guide, coordinate, and communicate the vision towards constructive change. (Kotter 2007) The leading organization and the Board decided to lead the campaign with the support of: ‘…expert advice on [their] strategy to influence government’. Financial resources of the leading organization enabled the hiring of an external consultant group with extensive experience leading advocacy campaigns for public and private sectors. This: ‘helped us to move forward a lot quicker with that expertise guiding us through the avenues that we could take.’ The leading organization campaign team, implemented the advice and strategy developed by the external consulting group. The consultants ‘helped us shape the campaign, shape the messaging and come up with the key messages and the whole look of the campaign.’ The group brought: ‘a long track record of work on political campaigns…’, experience relevant to the decision to work with them on the advocacy campaign.

A key decision at this point was to stay ‘solutions focused’, with a simple and clear message, which ‘cut through at a public sphere kind of level’. Reflecting their stated intent and responding to the urgency identified through the leading organization’s research and stakeholder communications, the campaign intent was expressed in the simple and blunt call: ‘get funding decisions down to 10 days….’ ‘creating a simple statement saying, surely we can do better.’ The 10 day target was informed by the timeframe that the NDIA committed to as part of its Participant Service Guarantee at one point. The NDIA backed away from this commitment when data was provided on the time taken for the NDIA to make decisions about SDA. The 10-day target was also informed by broader comparisons to the aged care system, where approval and placement processes tend to be faster than those experienced under the NDIS. For example, in 2017–8, the median elapsed time from approval to entry into permanent residential aged care was 34 days (Australian Government 2019). Additionally, among people with dementia transitioning from hospital to residential aged care, those flagged as ‘eligible and awaiting entry’ typically spent 13 days longer in hospital than those not awaiting placement (Australian Institute of Health and Welfare 2020).

It was important not: ‘to be a lone voice’, but to add ‘strength to the message if we had all the other organizations that were similarly frustrated.’ In order to do that ‘we had to assemble this really broad coalition, this alliance, in order to advocate from as broad a foundation as possible.’ The message was ‘personal and at an individual level’ so that it resonated with the general public, as the community was recognized as central to promoting change. This finding underlined the need to involve the general public: ‘because we’re affecting general public, providing more impetus for media, as well as activity and action from the government stakeholders.’ Thus, a list of organizations and allocated contacts were collated ‘based on who knew who….Yep, I know the CEO there. Yep, I know so-and-so there, …’, and those contacts were provided with campaign relevant resources they could post and share with their colleagues and clients.

### Form a strategic vision

This theme describes the process of developing the Campaign vision, direction and strategies. The vision was informed by the frustration of working with people with high support needs with an urgent need for housing while new disability housing stood vacant with a long and bureaucratic process required to get access to funding for housing and support through the NDIS. The issue identified was ‘not a lack of demand or a lack of supply, it is the slow—and often inaccurate—provision of housing and [funding] supports by the NDIA.’ (While this quote reflects the participant’s perspective, it is important to acknowledge that delays in housing and support provision are influenced by a complex interplay of factors, with the NDIA representing one significant component within a broader system.) The decision-making process and the reasons for final decisions needed to be better communicated. This part of the problem was described as ‘absolutely solvable. It simply requires the Federal Government to prioritize getting people with a disability the housing support they need.’

Initial meetings with experienced political lobbyists, informed ideas shaping an effective pre-election campaign and very specific asks to both sides of politics. The goal was to develop ‘something that we could clearly communicate but also potentially get a broad base of organizations and people with disability behind.’ The campaign needed a compelling message that resonated with a broad range of voters, and for a successful pre-election campaign, the leading organization had to demonstrate this issue could influence peoples votes.

The vision aimed to drive changes towards a ‘broader approach in terms of the messaging around NDIS decisions more broadly, not just the housing.’ A deliberate approach was this was not to be ‘a campaign to put [the leading organizations] name on anybody’s radar or anything like that. It was about getting the issue on the agenda and getting a commitment to the solution, rather than promoting [the lead organization]’.

The enacting of the vision had two important prerequisites: ‘not surprising the government and the Agency [NDIA]. …Then the second component was that we’d have others supporting it. We weren’t going to be a lone voice.’ This added strength to the message by involving ‘other organizations that were similarly frustrated.’ At the same time, the goal was not to create a hostile environment, with ‘efforts made to keep the cooperation strong and positive’, towards constructive change. For example, the leading organization developed a discussion paper that outlined potential strategies to improve NDIA decision-making timeframes for SDA. The paper summarized existing NDIA and hospital processes and proposed a series of solutions across four broad themes: enhancing the quality of evidence provided to the NDIA, streamlining home and living decision processes, strengthening the capacity and consistency of the home and living panel, and improving communication of panel decisions. Following engagement with the NDIA, the leading organization collaborated with the Occupational Therapy Association and the NDIA to support the development of tools aimed at improving the quality and consistency of evidence submitted by occupational therapists.

### Enlist a volunteer group

The fourth theme describes the recruitment and rallying of likeminded people around a common outcome. Building a coalition of committed participants was important to demonstrate the breadth of those with the same intent: ‘we had enough people on our side … (and show) the government that this is justified and what we’re saying is what others are also saying’. Creating a unified voice was a clear and deliberate strategy. Unlike many campaigns, this one involved a participation opportunity for all with an interest in health systems and services—including families, people with disability, and the general public—regardless of their role, and was designed to be attractive to many different organizations.

A list was compiled by the leading organization of ‘people in the housing sector, people in the disability sector, people that have similar objectives as we do, SDA providers as well. The health sector as well’. Forming an alliance at the start of the campaign was an efficient way of presenting a common voice across a variety of organizations: ‘there’s a group of probably six to eight organizations that have that (young people in nursing homes) at their core…. if you’re going to lobby government, you don’t want eight organizations going to the minister, you want to go once with all your brands at once and be aligned.’ Existing relationships within organizations with ‘larger and more reputable organizations we felt would add the most weight to the campaign’ built on past engagement with individuals and knowledge of who ‘would want to support it.’ aiming to get them on board quickly.

This initial list was extended ‘beyond that top tier group, we then moved down the list into [Electronic Direct Mail] EDMs. (An) email campaign saying, we are lobbying towards government and we want to ask for a decision to come down to 10 days, will you join us, will you join our alliance?’ The use of EDM marketing strategies enabled sending personalized emails to enhance connections and encourage participation. ‘The top 50, the top 100, organizations that also cared about this matter and [were asked] ..to join us to push the government to commit to Down to 10 Days’. Team members were assigned as key contacts for organizations, using provided template emails, phone calls, or personal contacts as they chose. Focusing on organizations: ‘we were really claiming the organization as a member, rather than the individual we were speaking to.’

The campaign reach grew to include over 130 organizations in the target sectors, who when agreeing to participate were offered formal alliance membership: ‘If you’d like to join our alliance send through your logo, you’ll be added onto our website and you’ll become one of the official alliance members.’ This adding of logos had a domino effect, with more organizations being recruited by either being incentivized or peer pressured into partaking, following the lead of bigger organizations. The importance of building numbers was key, ‘the more names we got, the more weight that that gave behind the campaign.’ There was a clear deadline with the full page advertisements in all of Australia’s largest newspapers listing campaign supporters.

### Enable action by removing barriers

This theme describes the development and implementation of processes to enable ready participation in the Campaign, with the aim of empowering stakeholders to engage and act to advance the common vision. This step supported the continued engagement of alliance partners further enhanced by strategies ensuring both consistent messaging and ease of action for those asked to promote the vision.

Activating the growing network of alliance partners amplified the key messages and content on social and digital platforms. Collectively, through all these organizations ‘you’re covering millions of Australians, easily millions of Australians’. One of the main things asked of Alliance members was promotion of the campaign through their own avenues: ‘through your LinkedIn, through your staff emails. We created a marketing kit for alliance members with suggestions of social media tiles that they could put out, facts related to the campaign that they could share. Specific wording that they could share.’ Templates were set up which ‘made it easy for them to be aware of the facts and figures and the people involved in the situations and to write contributing letters to all the stakeholders’. Other supportive actions included provision of curated letters for federal government politicians as well templates, images, videos, and all the lived experience curated by the leading organization. These resources could be used to support actions chosen by Alliance members to: ‘write it themselves or they can use what we’ve already created’, with the intention of ‘sending as many letters as possible for the awareness of that issue to grow amongst that government group.’ The provision of templates and social media tiles onto which organizations could put their own logo, was designed to encourage and start the process. ‘because we understand that not everyone has the time, resources to actually contribute.’

The ability to leverage data and the high-profile campaign was supported by paid advertising, encouraging media outlets to write stories on the issue; consequently pressuring politicians and generating public awareness. Being a member of the alliance meant being a part of impactful ‘front page news stories about slow decision-making’ which led to desired outcomes: ‘I know people at the NDIA who said we saw that story this morning and people were running around saying we need to do something’. The use of the media on behalf of the alliance was considered and deliberate with traditional media (such as TV and newspapers) used to influence politicians and the NDIA. This put politicians under pressure to make a statement on the issue. The external agency was pivotal in choosing the right media outlets.

Another option designed to support general public engagement, was a petition shared on members’ platforms. ‘Some of them (Alliance members) alone probably have 500 000 members or people that they work with. Some of the larger ones did share their material, you’d see immediate spikes in terms of the numbers coming through to sign’. These strategies supported participation, reducing time required and promoting ownership and action. As new members came on board, their memberships and actions were amplified, serving as a reminder for those who had not, to now adopt the media kit or letter.

### Generate short-term wins

There were many short-term wins, (some of which have already been described), as the campaign was tracked. Wins were celebrated a process that iteratively responded to achievements and changes, broadening emphasis and pivoting direction to best make use of the moment. Measurement of reach (e.g. social media) was a missing capability emerging during the campaign.

Most wins related to the continued building of Alliance members, petition signatures, mainstream media coverage and relationships or gains in the political arena. These were generally at either high level and politically significant, or more discreet like achieving a housing outcome for an individual. Regardless, as one interviewee noted: ‘even if only one person was helped by the campaign, that would be a win in itself’.

The campaign involved many different parts of the leading organization’s teams internally, including the government relations, executive, communications, storytelling, operations, and research teams. Teams would meet weekly to assess current status, what had been achieved, the numbers of petition signatories and alliance members, and the social media reach so far, each achievement: ‘mentioned here with this organization, on Twitter, or whatever it might be. That was always celebrated’.

Both the Government and the then Opposition acknowledged the issue, particularly the Shadow Minister, who chose to pick it up and run with it which was, ‘evidence of cut-through or success’. Significantly, the relevant opposition Minister was asked about the issue on a number of occasions, and in response ‘spoke in ways that essentially mirrored the language that we were putting forward’. The Opposition started talking publicly about delays in funding, specifically, ‘the thousands of people or NDIS participants stuck in hospital. That’s the cohort they focused on. That got a bit of traction. The Herald Sun published an article specifically talking about that. I think they got data from the government somehow about the number of people stuck in hospital waiting for funding. That directly tied into our campaign and what we’ve been saying’.

Public dissemination included sharing stories of people with disability on social media regarding their housing experiences. Stories highlighted that ‘you’ve just acquired a disability and you’re sitting in hospital, (in what) is probably one of the most vulnerable moments of your life’. The impacts of these individual actions are difficult to quantify, but they brought a human face of the problem to the public arena. These stories assisted wins at political levels, helping convince both politicians and the NDIA to both listen and hear the campaign messages. In response to actions, ‘in the third quarter of ‘22-’23—so this was probably two months after the campaign started—(NDIA) for the first time, included data on how long decisions were taking’ in their quarterly reports. This new table included notes and data about ‘this is how many decisions are taking this long’. Significantly. it also had notes identifying ‘this is an area that the agency is committed to improving, or something of that ilk’. All of which was a win, with new data being published, addressing issues related to the campaign.

### Consolidate gains and produce change

As the election was nearing, calls for politicians to respond to the Campaign vision were further strengthened by the increasing media coverage and supporters, in particular by the challenging nature of the stories and videos of those impacted by delays in decisions. Making their experience real and human was a powerful contribution, frequently evoking shock in response to what had previously been a ‘hidden’ issue. ‘I don’t think that publicly people were aware that that is actually the process. When people became aware that, oh wow, that is the process, that’s where the outrage and the push for government is, well, it can’t be that a person is in hospital waiting for a decision to be made over 300 days. That’s not right. It needs to change’.

The impact of these individual stories was carefully monitored, with considerations of audience and reach for placement, judging ‘the right time and the right place’ to favourably increase engagement. ‘We had stories in virtually every mainstream (medium)….. Everything from The Australian, to the ABC. There was a lot of awareness around the campaign’. Media coverage put pressure on the government, ‘(this) then affected the government because that became a thorn in their side’. Media interest was monitored and opportunities identified. While contemporary newspapers do not necessarily have big readerships they presented: ‘the opportunity to amplify those stories, and politicians as a group, take more notice of media, I think, in some ways, than the general public does’. Strategies targeted key points within the political cycle: ‘We placed newspaper ads during budget week. Because we knew that in that week those politicians and ministers would be reading the news at that particular time. We took out a full page spread, I think in two newspapers, with our campaign material and a list of all our alliance members at that point, to put it right under their nose’.

This approach of working with the various stakeholders rather than ‘taking pot shots at the NDIA’ enabled a constructive relationship—and as evidence accumulated providing pressure and ‘ample opportunities to engage on this issue’ constructive responses were provoked: ‘there is a significant following behind the campaign. That was brought to the attention of (Government Minister) and (opposition Minister) via those groups …. when they come to their round tables’. Further evidence of the cut-through of the campaign was increased engagement with the NDIA, including a visit from ‘the CEO… visited our offices at the Organization’s for a meeting specifically about this (issue)’.

### Institute change—anchoring new approaches in the culture

The last theme describes learnings, changes and outcomes, both internally and externally, as a result of implementing the campaign. The starting point was the recognition of the need to ‘Try something different. Something needed to change.’. This required a series of activities identified by participants as novel to the leading organization including building a team with internal and external participants with particular expertise; the deliberate recruitment of disability advocacy allies; and extended use of media—social and public.

Reflections on what worked and why included the difference between this and other campaigns. ‘We have run other campaigns, [independently without external consults] so the success of this one was because I think it was a multimodal campaign. It had lots of different parts, it wasn’t just this or this. It was everything combined’. While the challenges of reducing such a nuanced issue involving so many stakeholders, processes and funding systems and advocacy groups to a simple message were recognized, urgency and timeliness were powerful motivators. The simple data informed messages and aim ‘to try and actually instil support and understanding more broadly across (the population)—from a layman’s perspective’, was an effective approach. The use of media was both expedient and relatively cost effective, especially social media. Strategies adopted included a Google awareness campaign, so Google listings, and extensive use of EDMs, much of which has been described already. These initiatives were described as being implemented for the ‘first time at that level’, but as noted, the value of the approach was recognized and ‘having done something like that before, it could easily be activated again’.

The pre-existing relationships with Ministers and other Government officials was built on respect already in place for the quality of the work done by the leading organization, and that ‘it’s not an organization that willingly catastrophizes without a fact base behind it’. The leading organization had raised issues, shared data and proposed potential solutions during meetings prior to the commencement of the campaign. This provided a solid foundation for ongoing discussions and negotiations, initially informed by the overt ‘fair warning’ that action was needed with the rationale that ‘It’s not our first option, but we’ll do it, nothing's changing’. The ability to implement a public strategy if circumstances require, is a key change in culture and practice which may be employed where necessary.

Some challenges were identified which can be used to inform future campaigns. Most related to the expectations and activities of the members of the Alliance. Despite the provision of media kits including template letters to send to the government, ‘only a few organizations used their social media reach to support the campaign. A problem with bigger organizations was that they often already had their own campaigns running. But even just having their logos on the campaign gave it more weight and visibility’. What was noted to happen a lot was that ‘a lot of people would sign up as individuals because they couldn’t necessarily get their whole organization across the line, for example, if you worked in a public hospital’, which was really useful.

One of the challenges which was identified as a contributor to these issues was the speed of the campaign, necessitated by the upcoming election. ‘A longer lead time means you have a longer time to educate and a longer time to push material out there to get people on board your story….There’s more of that educational piece that you could have to bring on maybe other parties as well that we didn’t—weren’t planning on bringing on board….I think a longer lead time would’ve just meant a lot more promotion and promotional opportunities maybe’. However, it is important to highlight that the campaign was developed swiftly in response to consistent evidence of the government ‘kicking the can down the road’ and not making progress on the issue. Thus, in light of the approaching election, ‘[the organization] needed to change the tactic,’ with a public campaign being the route forward.

While a pre-to post sentiment analysis in the public would have been helpful, a heightened awareness for the issue has been noted as one of the campaign outcomes. Further, the NDIA and the government are more aware of the waiting times and people stuck in residential aged care and hospitals. The NDIA started reporting waiting times and the number of people waiting in hospitals in their quarterly reports, which increases transparency. Direct impacts of the campaign are difficult to prove, but many things changed which were seen as being influenced by the activity.

While it is not possible to determine the causal impact of the campaign on health system change, health system changes have subsequently occurred. The campaign aim was to reduce decision times—and the evidence is clear ‘basically, your decision timeline is moving downwards’, since the creation of the campaign…’. It's quite significant when you look at it from 12 months on. So, the fact is that they (NDIA)—12 months ago, or a bit more than 12 months ago now started reporting on it’, and, ‘I think we’ve seen a 50% reduction in the number of decisions taking more than 60 days, and a 100% improvement in the decisions taking under 30 days’. This reduction in timeframes has now been reported (Skipsey *et al*. 2023).

Getting Government to act was a major achievement, being able ‘to force the bear out of the hole, so to speak’, and regardless of what extent was causation or correlation this was likely the first time broad awareness was given to the fact that people were ‘given entitlements under the NDIA, but were effectively getting denied them because there was never any answer on anything they ever did in the slow turnaround times’. Having the now Minister taking the issue up quite personally led to very different outcomes, in response in part to having ‘everyone—all the people, all the organizations in the sector aligned around it, that it wasn’t going away, and it raised the general public awareness of the issue’.

While it is recognized that there is ‘still just a long way to go…we did effectively put pressure on government to improve their time frames and processes’.

## DISCUSSION

### Key campaign achievements and strategic foundations

The *Down to 10 Days* campaign aimed to address significant delays in NDIS processing times for housing and support decisions impacting the well-being and independence of people with disabilities. The process evaluation revealed several key findings. Firstly, the campaign effectively highlighted the severe delays in NDIS decisions, which left many individuals inappropriately in hospitals or aged-care facilities. Research delivered by the leading organization underscored the urgency, demonstrating processing delays as a critical barrier to suitable housing for people with disabilities. Secondly, formation of a strong coalition of over 130 organizations, enhancing the campaign’s credibility and reach. This coalition, although relatively passive, provided a united front advocating policy changes. Thirdly, strategic partnerships with expert campaign strategists, and key stakeholders in the disability and health sectors were crucial. Regular meetings with the NDIA and other governmental bodies helped maintain a cooperative rather than adversarial approach. The leading organization also produced a discussion paper outlining potential solutions for achieving faster and more accurate decisions on housing and support (Skipsey *et al*. 2023). Additionally, the campaign’s clear and simple message—reducing NDIS decision times to 10 days—was compelling and helped garner broad support. Finally, the use of multiple media channels and a well-organized petition gained significant public and stakeholder engagement, though reaching a wider public audience remained challenging.

### Systemic context: delays in care and housing transitions

Recent research on delayed transfers of care (DTOCs), or delayed discharges, identified their significant implications when patients are ready to leave hospital but are prevented by non-clinical reasons, such as delays in hospital processes, coordination issues, or lack of community care (Foster *et al*. 2022; Australian Medical Association 2023 ) In Australia, DTOCs have not been adequately addressed by national policy, leading to longer hospital stays, delayed surgeries, and hospital overcrowding, all exacerbating challenges faced by people with disabilities in need of timely housing decisions (Foster *et al*. 2022; Australian Medical Association 2023).

### Campaign impact

While causal attribution is not possible, several system-level changes occurred following the campaign that align with its aims and messaging. Notably, heightened public and political awareness of delays in hospital and aged care discharge for NDIS participants was achieved, with the NDIA subsequently incorporating hospital wait times into their quarterly reporting—enhancing transparency. Stakeholders also observed documented a marked reduction in decision timeframes pre campaign vs post campaign, with reports indicating a 50% decrease in delays over 60 days and a considerable increase in timely decisions (Skipsey *et al*. 2022, 2023). The campaign’s ability to unify sector voices and secure direct ministerial attention appears to have played a catalytic role in elevating the issue on the national agenda. While causality cannot be claimed, these shifts suggest the campaign contributed meaningfully to policy responsiveness and reinforced public accountability.

### Alignment with advocacy literature

This campaign aligns with existing literature highlighting advocacy’s role in addressing systemic barriers to housing and health equity for people with disabilities (Fisher and Purcal 2010). Previous research indicates that delays in bureaucratic processes significantly impact the well-being and autonomy of individuals with disabilities, which supports the campaign’s focus on timely decision-making by the NDIS (Skipsey *et al*. 2023). The necessity of a strong coalition for effective advocacy, highlighted by Moore *et al*. (2013), was evident in this campaign. The involvement of a broad range of organizations provided a robust platform for pushing policy changes. Additionally, the campaign’s strategy of leveraging public and political pressure aligns with successful public health advocacy strategies documented by Rosewarne *et al*. (2021).

### Implications for future advocacy campaigns

The implications of the *Down to 10 Days* campaign are multifaceted. It underscores the need for streamlined NDIS processes to ensure timely access to suitable housing for people with disabilities. Policymakers should consider reforms reducing bureaucratic delays and improving communication between the NDIA and stakeholders. The success in forming a coalition and gaining public support through media campaigns highlights strategies that can be replicated in future advocacy efforts. Rigorous process evaluation, with continuous assessment and adaptation based on feedback and emerging data, is crucial for advocacy success.

### Strengths and limitations

Several limitations were noted. The proximity of the federal election imposed a tight timeline, limiting alliance building and public engagement. While the campaign’s message was clear and compelling, it may not have captured the nuanced realities of all people with disability. The expectation for greater coalition member engagement was not fully realized, which may have impacted overall effectiveness. While the campaign gained attention and achieved short-term wins, we cannot say for certain that it directly caused the changes seen—like faster NDIA decisions or more transparent reporting—because there was no formal before-and-after study. Finally, the general public were not interviewed as part of this evaluation, limiting insight into broader community impact. Despite these limitations, the evaluation has several methodological strengths. An evidence-based process evaluation framework (Kotter’s Eight-Step Change Model [Kotter 2007]) was applied, and a hybrid deductive–inductive analysis approach ensuring both theoretical alignment and the capacity to capture context-specific insights was utilized. Triangulation of interviews and documents helped reduce bias and strengthen credibility. Finally, purposive sampling ensured that stakeholders with expert understanding of the issue and program were involved, ensuring informed knowledge of the campaign. These methodological features contribute to the relevance, and transferability of the evaluation findings.

## CONCLUSION

The *Down to 10 Days* campaign provides a valuable case study in public health advocacy, particularly in the disability sector. By highlighting systemic issues and mobilizing a broad coalition, the campaign achieved significant short-term wins and laid the groundwork for ongoing advocacy efforts. Future campaigns can build on this experience to further advance the rights and well-being of people with disability, towards ensuring full inclusion and social participation.

## Supplementary Material

daaf082_Supplementary_Data

## Data Availability

The data supporting this study, including internal documents and interview transcripts, are not publicly accessible. As identifiable information was collected, participants provided consent for the raw data to be available to the research team. For any inquiries regarding access to the data, please contact Dr Ali Lakhani (a.lakhani@latrobe.edu.au).

## References

[daaf082-B1] Australian Government . *Unmet Needs In Aged Care: How Long did Australians Wait for Aged Care Services? Australian Institute of Health and Welfare*. 2019. https://www.gen-agedcaredata.gov.au/getmedia/2f3bebcc-e230-4249-bfc6-73e622a1e9aa/Unmet-needs-in-aged-care-How-long-did-Australians-wait-for-aged-care-services.pdf (22 February 2025, date last accessed).

[daaf082-B2] Australian Institute of Health and Welfare . *Transitions to residential Aged Care After Hospital for People with Dementia*. 2020. https://www.aihw.gov.au/reports/dementia/transitions-to-aged-care-after-hospital-dementia/contents/summary (22 February 2025, date last accessed).

[daaf082-B3] Australian Medical Association . *Hospital Exit Block: A Symptom of a Sick Health System*. 2023. https://www.ama.com.au/sites/default/files/2023-02/Hospital%20exit%20block%20-%20a%20symptom%20of%20a%20sick%20health%20system_Final.pdf (22 February 2025, date last accessed).

[daaf082-B4] Bhattarai JJ, Bentley J, Morean W et al Promoting equity at the population level: putting the foundational principles into practice through disability advocacy. Rehabil Psychol 2020;65:87–100. 10.1037/rep0000321 32297777 PMC7285891

[daaf082-B5] Brolan CE, Boyle FM, Dean JH et al Health advocacy: a vital step in attaining human rights for adults with intellectual disability. J Intellect Disabil Res 2012;56:1087–97. 10.1111/J.1365-2788.2012.01637.X 23106752

[daaf082-B6] Capitão C, Martins R, Feteira-Santos R et al Developing healthy eating promotion mass media campaigns: a qualitative study. Front Public Health 2022;10:931116. 10.3389/fpubh.2022.931116 35968460 PMC9372615

[daaf082-B7] Cubis L, Ramme RA, Roseingrave E et al *Evaluating the Discharge Planning Process: Barriers, Challenges, and Facilitators of Timely and Effective Discharge for People with Disability and Complex Needs*. 2022. https://assets.summerfoundation.org.au/pdf_offload/2022/08/Evaluating-the-discharge-planning-process-report-May-2022.pdf (22 February 2025, date last accessed).

[daaf082-B8] Davies R, Dart J. The 'most significant change' (MSC) technique: a guide to its use (Version 1.00). 2005. https://ahi.sub.jp/eng/wp-content/uploads/2022/02/MSCGuide-1.pdf (22 February 2025, date last accessed).

[daaf082-B9] Fereday J, Muir-Cochrane E. Demonstrating rigor using thematic analysis: a hybrid approach of inductive and deductive coding and theme development. Int J Qual Methods 2006;5:80–92. 10.1177/160940690600500107

[daaf082-B10] Fisher KR, Purcal C. Effective personalised housing support for people with disabilities–case study analysis. Aust J Soc Issues 2010;45:527–42. 10.1002/j.1839-4655.2010.tb00196.x

[daaf082-B11] Flavel J, Freeman T, Musolino C et al Health promotion and the need to accelerate advocacy for health equity. Health Promot Int 2024;39:daae040. 10.1093/heapro/daae040 38722019

[daaf082-B12] Foster M, Henley G, Griffiths A et al Planning with care complexity: factors related to discharge delays of people with acquired neurological disability. Health Soc Care Community 2022. 10.1111/hsc.13912 PMC10087249 35880633

[daaf082-B13] Freudenberg N, Bradley SP, Serrano M. Public health campaigns to change industry practices that damage health: an analysis of 12 case studies. Health Educ Behav 2009;36:230–49. 10.1177/1090198107301330 18077655

[daaf082-B14] Jaiswal A, Gupta S. Advocacy campaign for the rights of people with disabilities: a participatory action research within a community-based rehabilitation project in Vangani, Maharashtra. Disabil CBR Inclusive Develop 2017;27:76–92. 10.5463/DCID.V27I4.529

[daaf082-B15] Kotter JP . Leading change: why transformation efforts fail. In: *Museum Management and Marketing.* Routledge, 2007, 20–9.

[daaf082-B16] Lakhani A, McDonald D, Zeeman H. Perspectives of self-direction: a systematic review of key areas contributing to service users’ engagement and choice-making in self-directed disability services and supports. Health Soc Care Community 2018a;26:295–313. 10.1111/hsc.12386 27641903

[daaf082-B17] Lakhani A, McDonald D, Zeeman H. Perspectives of the national disability insurance scheme: participants’ knowledge and expectations of the scheme. Disabil Soc 2018b;33:783–803. 10.1080/09687599.2018.1442321

[daaf082-B18] Mackenzie-Stewart R, Khalil H. Evaluating Health and Social Care Programs. Leading in Health and Social Care. Griffith University, 2023.

[daaf082-B19] Moore M, Yeatman H, Pollard C. Evaluating success in public health advocacy strategies. Vietnam J. Public Health 2013;1:66–75.

[daaf082-B20] Morgan H . Conducting a qualitative document analysis. Qual Rep 2022;27:64–77. 10.46743/2160-3715/2022.5044

[daaf082-B21] National Disability Insurance Agency. About the NDIS . 2025. https://www.ndis.gov.au/about-us (22 February 2025, date last accessed).

[daaf082-B22] Office of the High Commissioner for Human Rights . International covenant on economic, social and cultural rights. 1976.

[daaf082-B23] Q. S. R. International Pty Ltd . *NVivo (Version 12)*. *In [Computer software]. QSR International*. 2020. https://www.qsrinternational.com/nvivo-qualitative-data-analysis-software/home (22 February 2025, date last accessed).

[daaf082-B24] Rosewarne E, Moore M, Chislett W-K et al An evaluation of the Victorian salt reduction partnership’s advocacy strategy for policy change. Health Res Policy Syst 2021;19:100. 10.1186/s12961-021-00759-1 34266477 PMC8281636

[daaf082-B25] Samuels-Dennis J, Xia L, Secord S et al Health advocacy project: evaluating the benefits of service learning to nursing students and low income individuals involved in a community-based mental health promotion project. Int J Nurs Educ Scholarsh 2016;13:97–108. 10.1515/IJNES-2015-0069 27705899

[daaf082-B26] Skipsey M, Winkler D, Cohen M et al Housing Delayed and Denied: NDIA Decision-Making on Specialist Disability Accommodation. Sydney: Public Interest Advocacy Centre and Housing Hub, 2022.

[daaf082-B27] Skipsey M, Mulherin P, Efstathiou M et al *Data Update: NDIA Decision-Making Timeframes on Specialist Disability Accommodation*. 2023. https://www.housinghub.org.au (22 February 2025, date last accessed).

[daaf082-B28] United Nations General Assembly . *Universal Declaration of Human Rights.* Vol. 3381. Department of State, United States of America, 1949.

[daaf082-B29] United Nations General Assembly . Convention on the rights of persons with disabilities. Ga Res 2006;61:106.

[daaf082-B30] US Department of Health and Human Services . *Data Collection Methods for Evaluation: Document Review*. 2018. https://www.betterevaluation.org/tools-resources/data-collection-methods-for-evaluation-document-review (11 June 2025, date last accessed).

[daaf082-B31] World Health Organization . *Ottawa Charter on Health Promotion*. 1986. https://www.who.int/teams/health-promotion/enhanced-wellbeing/first-global-conference (22 February 2025, date last accessed).

[daaf082-B32] Zhao X . Health communication campaigns: a brief introduction and call for dialogue. Int J Nurs Sci 2020;7:S11–5. 10.1016/j.ijnss.2020.04.009 32995373 PMC7501494

[daaf082-B33] Zoom Video Communications, I . *Zoom (Version 6.3.10) [Computer software]*. 2025. https://zoom.us (22 February 2025, date last accessed).

