# Supplementary material for: Inclusive housing for people with disability: process evaluation of the ‘Down to 10 Days’ campaign
Source: Health Promot Int. 2025 Jun 18;40(3):daaf082. doi: 10.1093/heapro/daaf082 (PMC12203791; doi:10.1093/heapro/daaf082)
Supplement: daaf082_Supplementary_Data [file daaf082_supplementary_data.docx]

**Supplementary File - Campaign Timeline Overview**

| **Phase**  **Month** | **Description** |
| --- | --- |
| **Pre-Campaign Phase** | |
| **July 2018** | Initiated a project to measure hospital discharge timeframes. |
| **July 2021** | Initiated collaboration with a public advocacy group. |
| **Campaign Phase** |  |
| **November 2021** | Presented a strategic board paper to discuss approaches for increasing project traction. Initial discussions with prospective campaign leadership, exploring the potential to leverage an election period for advocacy. |
| **December 2021** | Workshop held with key stakeholders to brainstorm ideas for informing Federal policy regarding disability support. Communicated new research findings to Federal representatives and began collaboration with a market research firm to guide campaign messaging. Introduced a preliminary strategy to the board, involving market research, strategic analysis, and proposed budgetary support. |
| **January 2022** | Commenced campaign-specific research to inform advocacy strategy. Developed a background paper to identify existing opportunities, relationships, and preliminary campaign strategy. |
| **February 2022** | Presented a pre-election campaign strategy to the board, with emphasis on advocacy and engagement with external research and consulting firms. |
| **March 2022** | Media plan and weekly campaign meetings commenced. Campaign launched with a media release and a direct communication to government leadership outlining campaign objectives. |
| **April 2022** | Government representatives announced measures aligning with campaign objectives, including reduced wait times for support access. Ongoing updates and media coverage emphasized campaign traction and public awareness. |
| **May 2022** | National media coverage highlighted the impact of delayed decision-making on hospital discharge times. Established working groups with national disability authorities to continue improving support systems. |
| **June – August 2022** | A series of focused meetings with disability agency advisors facilitated working group efforts to address policy challenges identified by the campaign. |
| **October 2022** | Additional media coverage documented ongoing barriers, reinforcing the need for continued policy change. |
